# Supplementary material for: Predicting monoclonal antibody binding sequences from a sparse sampling of all possible sequences
Source: Commun Biol. 2024 Aug 12;7:979. doi: 10.1038/s42003-024-06650-3 (PMC11319732; doi:10.1038/s42003-024-06650-3)
Supplement: Supplementary file 2 — Supplemental Material [file 42003_2024_6650_MOESM2_ESM.pdf]

Predicting Monoclonal Antibody Binding Sequences from a Sparse Sampling of All Possible Sequences

Supplementary Material

Pritha Bisarad[1,2,5,7,+], Laimonas Kelbauskas[3,9,+], Akanksha Singh[1,2,6,+], Alexander T. Taguchi[4], Olgica Trenchevska[8], Neal W. Woodbury[1,2,3,*]

[1]School of Molecular Sciences, Arizona State University, Tempe, Arizona, United States of America, [2]Center for Innovations in Medicine, Biodesign Institute, Arizona State University, Tempe, Arizona, United States of America, [3]Center for Molecular Design and Biomimetics, Biodesign Institute, Arizona State University, Tempe, Arizona, United States of America, [4]iBio Inc., San Diego, California, United States of America, [5]Pediatric Movement Disorders Program, Division of Pediatric Neurology, Barrow Neurological Institute, Phoenix Children's Hospital, Phoenix, Arizona, United States of America , [6]Prellis Biologics Inc., Berkeley, California, United States of America, [7]Department of Child Health, University of Arizona College of Medicine-Phoenix, Phoenix, Arizona, United States of America,[8]Cowper Sciences Inc., Chandler, Arizona, United States of America, [9]Biomorph Technologies, Chandler, Arizona, United States of America

[+]contributed equally
*corresponding author

Author emails:
NWW*:nwoodbury@asu.edu

PB: pbisarad@arizona.edu
LK: Laimonas.Kelbauskas@asu.edu
AS: akanksha9203@gmail.com
ATT: alex.taguchi@ibioinc.com
OT: olgica.trenchevska@cowpersci.com

Arrays and Data Quality.

Peptide microarrays containing diverse peptides were synthesized at Cowper Sciences, Inc., (Chandler, AZ) as discussed in the main text. After completion of synthesis, each wafer was diced into 13 slides (25x75mm) and 4 MALDI-MS arrays. Slides were further subjected to a standard chemical cocktail to remove side chain protecting groups and stored under nitrogen prior to use. Synthesis verification was performed by high-resolution Matrix-Assisted Laser Desorption Ionization Mass Spectrometry (MALDI-MS), using a previously described protocol.[1] Briefly, N-termini of peptides are labeled with TMPP (tris(2,4,6-trimethoxyphenyl) phosphine) to provide net positive charge, followed by selective chemical cleavage from the surface using gaseous ammonia. Alpha-cyano-4-hydroxycinnamic acid (CHCA) is then applied to the array surface using an automated sprayer (TM-Sprayer, HTX Technologies, LLC). MALDI mass spectra are acquired from each MALDI feature (representing each amino acid monomer and/or peptide) on an Autoflex Speed MALDI MS (Bruker Daltonik). Synthesis is verified when desired peptides are identified on the expected *m/z*, with a set tolerance of 0.5Da and minimum signal-to-noise (S/N) of 3.

Fig. S1 shows the Pearson correlation coefficient between the mAb's $\log_{10}$ binding values at each concentration and every other mAb's $\log_{10}$ binding values at each concentration. The four

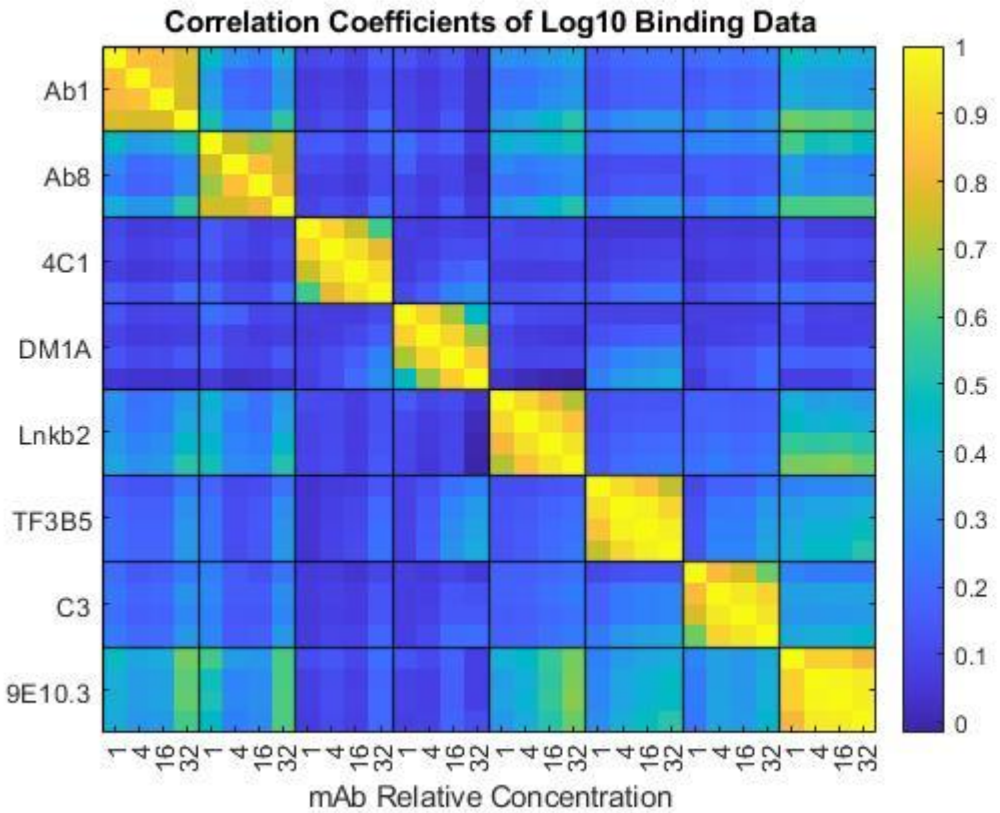

Figure S1. Pearson correlation coefficients between the linear binding values of array data after averaging replicates. For each monoclonal antibody, there are four concentrations (in increasing order, 0.125 nM, 0.5 nM, 2.0 nM, 8.0 nM).

replicates of each mAb and each concentration were averaged prior to calculating the correlation coefficient. The data is organized from the lowest concentration (0.125 nM) to the highest concentration (8 nM) for each mAb. As one moves along the diagonal, one can see that different concentrations of any particular mAb are generally similar, and different mAbs are quite dissimilar from each other. No data was excluded from the analysis.

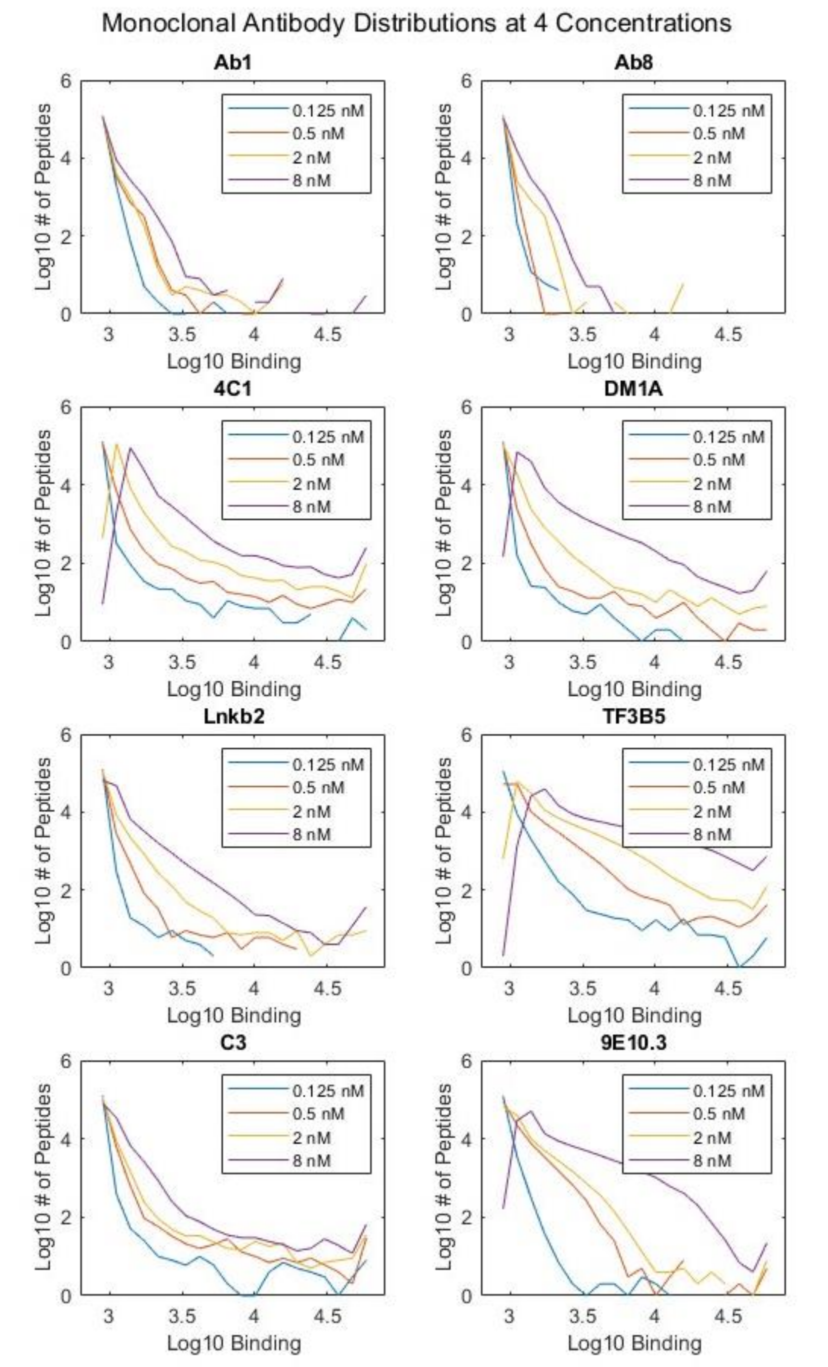

Figure S2. Log_{10} binding distributions of each monoclonal antibody at four concentrations.

Distributions. Fig. S2 shows the distributions of each of the datasets (each mAb at each concentration). Note that both the frequency scale (y-axis) and the binding value (x-axis) are log_{10}; binding is given in log_{10} fluorescence counts. The background binding of the labeled secondary antibody to the arrays is ~850 counts. There is considerable variation in the number of high binding value sequences on these arrays, with both Ab1 and Ab8 having very few sequences that bind with values above 3,000 counts (~3.5 on the log scale) and TF3B5 having hundreds of sequences with values that saturate the detector at 65,536 counts at the highest concentration (4.82 on a log_{10} scale). The binding values of the mAbs to their cognate sequences are not shown, but with the exception of 4C1, the signal from the cognate sequence is saturating even at 0.125 nM for those mAbs that had a cognate sequence synthesized on the array (Ab1, Ab8, 4C1, DM1A, Lnkb2).

The distributions give an immediate indication of the specificity of each mAb. Since the binding data is from peptides sampled nearly randomly over all of the 16 amino acid sequence space ($16^{10} = 10^{12}$ sequences) without regard for any specific biological relevance, this platform gives a simple empirical comparison of specificity in an unbiased dataset. In general, the vast majority of the binding for all the mAbs is near the background level and in the case of Ab8, there is almost no binding more than a few times the background level. As can be seen in Fig. S1, the correlation between binding of different mAbs is generally low, presumably due to very different interactions and sequence requirements. Thus, what one learns about binding of one mAb is unlikely to be useful in characterizing the binding of another. For this reason they were modeled separately.

Number of Sequences Above the Median for Each mAb

Table S1 shows the number of sequences for each mAb at each concentration that was at least 2 standard deviations above the median value of binding for all sequences for that mAb and concentration. The standard deviation was calculated for each peptide, each mAb and each concentration from the four experimental replicates.

Table S1: Number of Peptide Sequences >2 Standard Deviations Above the Median Binding Value

	Number of Sequences Above the Median			
**mAb**	**0.125 nM**	**0.5 nM**	**2 nM**	**8 nM**
**Ab1**	3	23	89	195
**Ab8**	0	1	2	9
**4C1**	89	123	364	1146
**DM1A**	19	69	321	101
**Lnkb2**	30	65	116	69
**TF3B5**	208	584	8428	7323
**C3**	29	48	178	228
**9E10.3**	28	111	5	13393

The numbers of mAbs above background by two standard deviations does not always grow in the way one might expect with concentration. This is for two reasons. First, there is a general increase in the median value as the concentration increases. Thus, the value being compared to is increasing. Second, if there happens to be an array that is uniformly scaled a bit higher or lower than the other replicates, that results in a large standard deviation which also causes the data to not exceed the 2 standard deviation threshold.

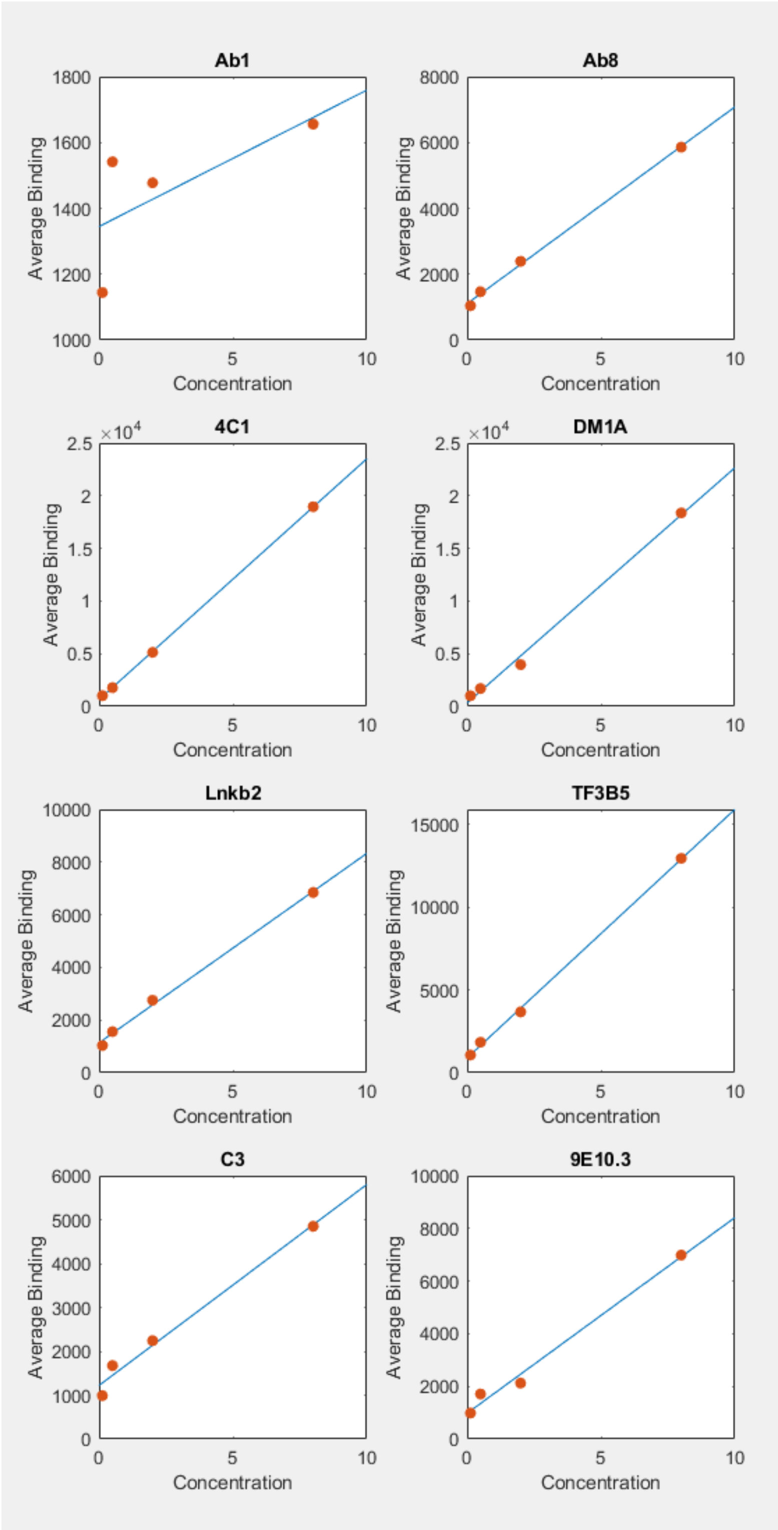

Figure S3. The average binding of mAbs to peptides on the array vs. concentration for all peptides that do not saturate at any concentration.

Linearity with concentration. In order to take into account the fact that at higher concentrations detector saturation occurs for some peptide binding, the points that saturated at one concentration are assumed to increase linearly with concentration at higher concentration. Fig. S3 justifies that assumption looking at the overall linearity of binding as a function of concentration for each of the mAbs. In each case, the average binding of all peptides that do not saturate at any concentration is plotted, showing that there is a linear dependence of binding on concentration at least within the dynamic range of the detector. Thus, the assay itself is very linear with concentration.

Cross validation study of binding prediction. Fig. S4 shows a 10-fold cross validation study of sequence-specific binding prediction models for each mAb as a density scatter plot[2]. Here the $\log_{10}$ predicted binding value is plotted against the $\log_{10}$ measured binding value. As described in the main text and above, saturating points were extrapolated based on concentration, which is why

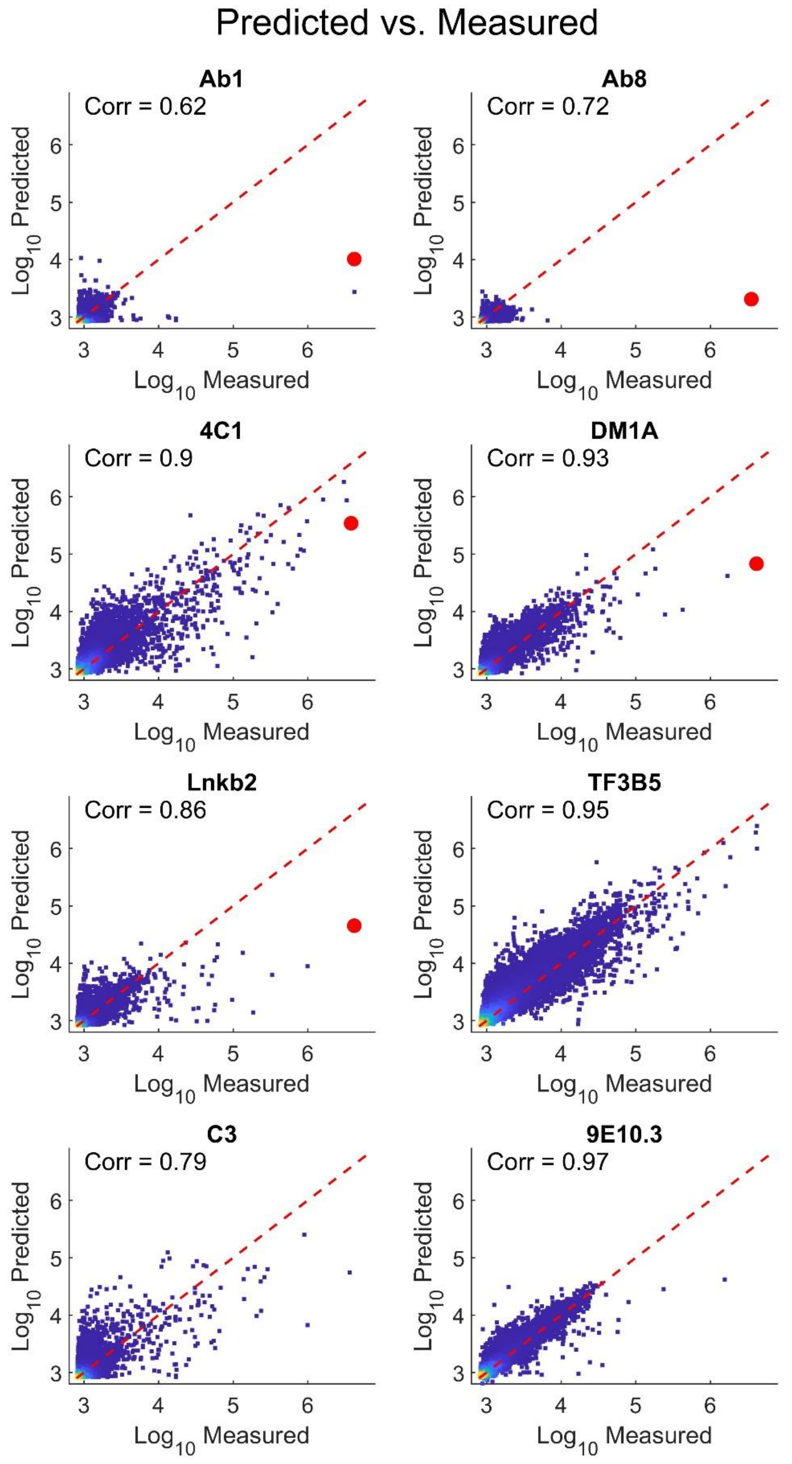

Figure S4. Density scatter plot of $\log_{10}$ predicted values of the sequence dependent binding vs. the $\log_{10}$ sequence dependent binding determined from the values measured on the array. Red dots are the cognate sequences. Only 100,000 randomly picked data points are shown for each mAb of the ~500,000 total points across all concentrations.

there are $\log_{10}$ binding values >4.82. 90% of the peptide-binding pairs (randomly selected) were used to train the model in each case and 10% were used as the test set. This was done for all ten possible 90:10 combinations so that all peptides were ultimately included in the test set. Then the entire prediction was repeated 10 times and averaged. Fig. S4 plots 100,000 predicted test binding values against the measured values. The displayed values were randomly chosen from all 4 concentrations modeled. It also shows, as a red dot, the 8 nM predicted and measured value of each mAb cognate sequence when that value is present on the array. Note that the predicted value of the cognate is generally lower than the measured value, particularly in cases like Ab1 and Ab8 where there are very few peptides that bind strongly. However, even though the value predicted for the cognate is generally lower than its measured value, its rank remains high among the 100,000 points plotted. Note also that the cognate prediction is being compared to sequences of many lengths (the array peptides range from 5 to 11 residues), rather than only to sequences of the same length. The length need not be exactly the length of the cognate sequence as long as it contains the cognate sequence, but both the predictions and

the measured data tend to show higher binding for longer peptides. Thus, when looking, for example, at binding to a protein, one would want to tile the protein sequence as even length pieces for an accurate comparison of different regions of the protein.

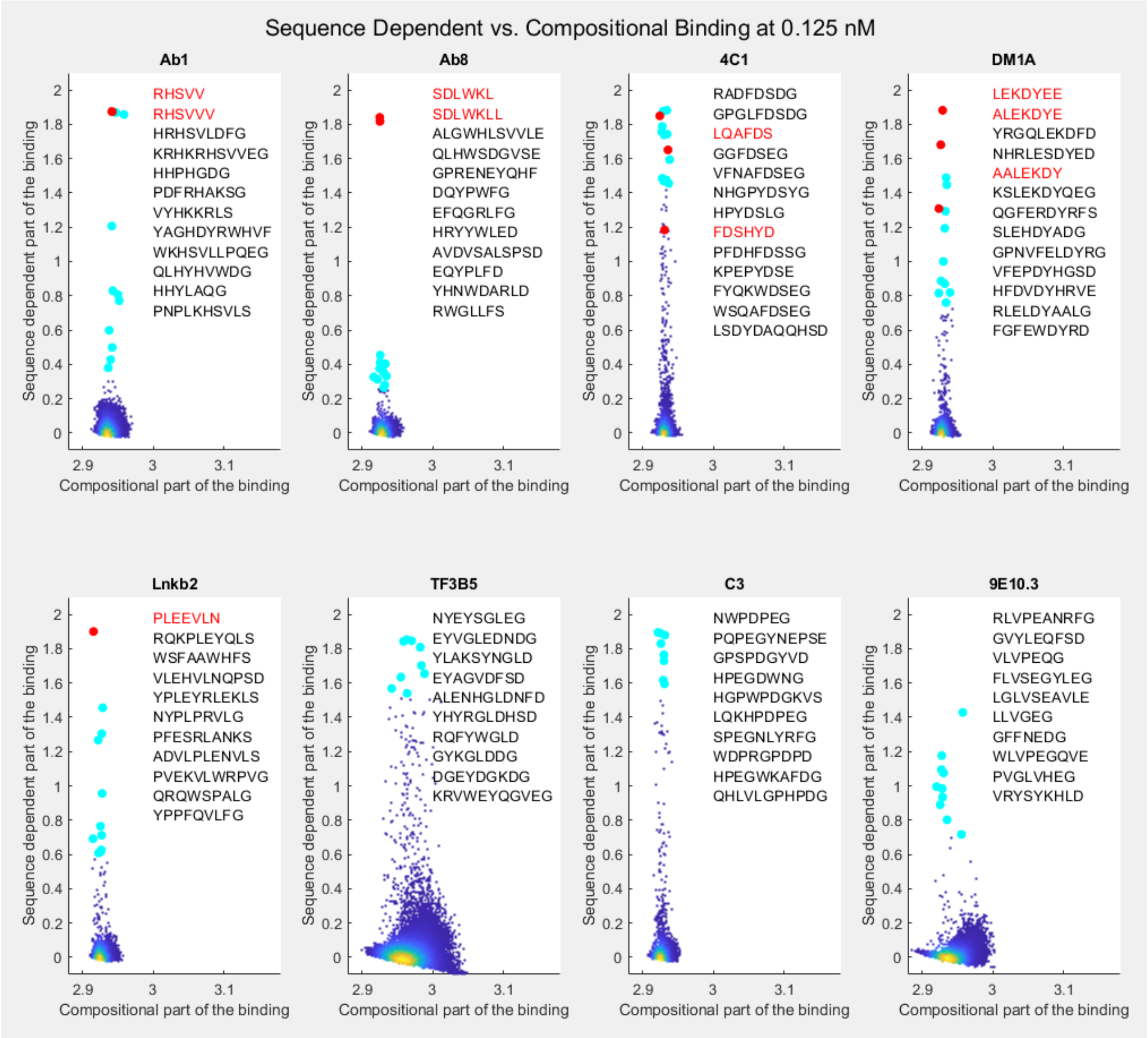

Figure S5. Sequence-dependent binding vs. compositional binding for each of the mAbs at 0.125 nM concentration. Each sequence is plotted as a scatter plot. Also plotted for reference are the cognate sequences that are present on the array (red circles). The top ten non-cognate sequences from the array are shown as light blue circles. Both the cognate epitope sequences (if present on the array) and the top 10 binding array sequences are listed for each mAb.

Compositionally Dependent Binding. Fig. S5 shows scatter plots of sequence-dependent binding (y-axis) vs. compositional binding (x-axis) determined as described in Methods of the main text. Basically, a set of coefficients relating the number of each amino acid in the peptide to the binding of that amino acid is determined in a linear fit (there is also a bias term). The amount of binding that can be described this way is subtracted from the total binding (subtraction

is performed after taking the log_{10}). In every case but 4C1, when the cognate epitope sequence in present on the array (shown as red points) it represents the strongest sequence-specific binding (the cognate epitope sequences for TF3B5, C3 and 9E10.3 were not present on the array). Most of the mAbs have a very narrow range of composition-dependent binding. In other words, the only term in the linear fit that was really significant in most cases was the bias term describing the background binding. Only a small fraction of the binding distribution (0.05 on the log_{10} scale) could be explained by composition alone. The two exceptions to this rule are TF3B5 and 9E10.3, both of which show a distribution of compositional log_{10} binding values >0.1. Even for these two mAbs, however, compositional binding is still modest compared to sequence specific log_{10} binding of 1.5-2. The overall amount of sequence specific binding among the array peptides varies greatly between mAbs. Ab1 and Ab8 have the least number of sequence-specific binding sequences. Ab8 in particular is devoid of any sequences that bind with sequence-specific binding values above 0.5, while Ab1 has only 6 such sequences. In all other mAbs, at least the top 10 sequence-specific binding sequence values (light blue points) are higher than 0.5. TF3B5 is at the opposite end of the spectrum, having about 100 sequences that show substantial sequence specific binding, including a number that saturate the detector (~1.9 on the y-axis). Other mAbs are between these extremes. 4C1 is the one case where none of the sequences synthesized on the array to represent its cognate epitope sequence are the highest binding sequences. This suggests that at least in the context of binding to a short linear sequence attached to an array surface, this mAb may be less specific than the other mAbs; there are apparently multiple different sequences that bind this mAb about as strongly as the cognate epitope sequence. Note, however that at least in terms of prediction based on the neural network models, adding additional amino acids from the binding region of the antigen increases binding of 4C1 (see below).

Also shown in Fig. S5 are the ten highest binding sequences on the array (in black type) as well as the sequences of the cognate epitope representations considered (in red, only present for those mAbs where they were measured on the same array as the rest of the data). As seen in Fig. 1 of the main text, in every case but Ab8, at least several of the top ten sequences are clearly related to the cognate epitope sequence. In the case of Ab8, the relationship is more difficult to discern, though one can see a similarity in the particular amino acids used. The general similarity of some of the stronger sequence-dependent binding sequences to the corresponding cognate epitope suggests that sequence-specific information is indeed available on the array. It is important to remember that in this work, the epitopes are taken out of their folded protein environment. This may well decrease the binding value and specificity.

Ranking based on binding. Ranking of predicted cognate binding values is determined based on binding at the four concentrations of mAb. Each concentration was separately ranked and then the lowest rank of the four was assigned to each peptide. This can result with two peptides having the same rank, and thus the peptides were reranked so that the rankings went from 1 to N were N is the total number of peptides. The same algorithm is used for all of the million random peptides and the cognate, so there is no bias towards the cognate sequence. The reason this maximum rank approach was used is that different peptides lie in different parts of the dynamic range of the binding measurement. This was the least biased approach to letting the data decide what concentration to use for each peptide.

Top Random Sequences Predicted

The top 20 ranked sequences predicted in the 1 million sequence random library for each of the mAbs is given in Table S2. Note that the mAb cognate sequences are included as reference at the top. Again, one can generally pick out a commonality in sequence among them. In some instances, most notably Ab8, there are also unrelated sequences. For Ab8, those tend to be highly positively charged sequences. Interestingly, the N-terminus of the P53 antigen that Ab8 was raised against has a very highly positively charged region.

Table S2: Top 20 Sequences Predicted from Random Library of 1 Million

Rank	Ab1	Ab8	4C1	DM1A	Lnkb2	TF3B5	C3	9E10.3
Cognate	***RHSVVVP***	***SDLWKLL***	***QAFDSHY***	***LEKDYEE***	***PLEEVLN***	***PEYLGLD***	***SLPNPEG***	***KLVSEED***
1	HKRHSVL	RRYRRGR	GPFDSLG	HLEADYA	PPLELVL	PEYLGLD	WFPNPEG	FLVPEWR
2	RRHSVVD	KKSGLGK	QPFDSYG	VFEKDYL	PLERVLD	HEYKGLD	SSPSPEG	LLVGEKH
3	HRHRVRD	LKWGLGK	LQPWDSH	FERDFFD	RPLQQVL	WEYGGLD	FWPNPEG	LLVPEWL
4	HRSSVLF	VRRRYRR	FGYDSNG	QLERDYH	RPLQRVL	EYWGLDN	FSPNPEG	RLVPENY
5	HRHWVVD	LHSDLLK	LGYDSFG	FEYDYPE	PLEYQLG	SDYWGLD	SSNPWPE	GVLVPEQ
6	HRHSFVP	EPGWKGY	APFDSDG	SHFEADY	PFEKPLR	GEYVGLE	GPGPWPE	FLVPERW
7	HRHHVVK	YSRRRRR	YQPYDSV	RFEVDYE	PLEAKLL	HEYLGLN	GLNPWPE	WLVSEAL
8	RHSVVAV	KSDFGKL	AAYDSHY	GSLEVDY	YPFEKLL	DQYWGLD	WPSPDGF	LLVPELW
9	VRHSVLH	NDLGKLK	PAGYDSW	YQFERDF	PLFEPLR	EYSGLNS	YYSPWPE	GLVSEGH
10	HHHHHHL	RHRKRRR	FDWDSQG	FDWDYHD	HPFESVL	QSEYSGL	WPYPEGY	LLVPESY
11	RRHSVHQ	FWRRRRR	AHYDSHY	GFERDYL	PFEKLRR	RSEYLGL	GWPWPEG	GLVPEWE
12	HKHSVVG	RRYRRRW	ASFDSYG	LEWDYDP	PLFEKLS	FEYLGLE	FSRPDPD	LRLVPEA
13	HKRHSHL	LLGLLKK	PGFDSVG	FEKDFDY	PPLEQQL	SYAGLDH	YHKPWPE	FLVAEGS
14	HPHSVLS	RHRRRRH	EPFDSHY	HNFEYDF	PFEVWLK	SYHGLDS	WNGPHPD	YLVGELN
15	HRHSEHV	LLKGLLK	YDFDSNG	PSFEWDY	VLENVLR	NSYLGLD	WWPPHPD	VLVPVAV
16	HSVLDPG	HRRRRLR	DSYDSYG	KVLERDF	RRYPPPL	YEYSGLK	FNSPNPE	QLVSEGF
17	HRHSSPK	RYRRRYY	LKPYDSV	DLEKDYE	PLEFYLA	YPEYPGL	HPHPDGF	WLRPEWW
18	RKSVVFF	RRRHQRR	SHFDSFG	NSFDKDY	PLEEHLK	SAEYPGL	WSGPWPW	FLVPEHE
19	VHRHSVQ	WRPRRRR	WREPWDS	QLELDYA	VLEKLLH	KEYHGRD	HPHPDGN	FLVGEWG
20	KRRSVLF	RRYYRPR	HGFDSRW	WLEWDYV	PFEVLFK	DYRGLDW	FPGPNPD	KLVPEPF

Table S3 Results from Full Model After Training with Randomized Array Sequence Order

mAb	Rank
**Ab1**	420,000 ± 170,000
**Ab8**	140,000 ± 40,000
**4C1**	310,000 ± 130,000
**DM1A**	300,000 ± 130,000
**Lnkb2**	370,000 ± 150,000
**TF3B5**	420,000 ± 130,000
**C3**	610,000 ± 100,000
**9E10.3**	530,000 ± 90,000

Randomized Sequence Order Control
Table S3 shows the result of training on a dataset (using the full model) in which the sequence order is randomized relative to the order of the binding values from the array. As expected, after averaging results across multiple training runs, the values approach the rank of 500,000 (50%).

Varying the length of the antigen sequence used as the cognate sequence. In order to better understand both the ability of the model to work with different lengths of sequences and to understand to what extent sequence context in the cognate region of the antigen matters in the modeling, model predictions similar to those of the full model in Table 2 of the main text were repeated for sequence regions between 6 and 10 residues, where the longer sequences were taken from the known binding sequence region of the

Table S4: Antigen sequences used for rank prediction at various lengths

mAb	6 mers	7 mers	8 mers	9 mers	10 mers
**Ab1**	RHSVVV	RHSVVVP	FRHSVVVP	FRHSVVVPY	FRHSVVVPYE
**Ab8**	SDLWKL	SDLWKLL	SDLWKLLP	SFSDLWKLL	SFSDLWKLLP
**4C1**	AFDSHY	QAFDSHY	LQAFDSHY	LQAFDSHYD	SLQAFDSHYD
**DM1A**	LEKDYE	LEKDYEE	LEKDYEEV	ALEKDYEEV	ALEKDYEEVG
**Lnkb2**	PLEEVL	PLEEVLN	KPLEEVLN	PLEEVLNLA	PLEEVLNLAQ
**TF3B5**	EYLGLD	PEYLGLD	NPEYLGLD	NPEYLGLDV	NPEYLGLDVP
**C3**	PNPEGR	SLPNPEG	PNPEGRYS	PNPEGRYSF	PNPEGRYSFG
**9E10.3**	KLVSEE	KLVSEED	KLVSEEDL	KLVSEEDLL	QKLVSEEDLL

Table S5: Ranks out of 1 million random sequences[1] of the sequences in Table S4

mAb	6 mers	7 mers	8 mers	9 mers	10 mers
**Ab1**	4.0 ± 0.6	3.0 ± 0.7	2.6 ± 0.4	15 ± 2	25 ± 4
**Ab8**	45 ± 10	7.9 ± 1.2	21 ± 7	68 ± 8	42 ± 16
**4C1**	22 ± 3	15 ± 1.5	5.4 ± 0.7	6.6 ± 0.7	3.4 ± 0.7
**DM1A**	6.6 ± 0.9	2.2 ± 0.5	1.4 ± 0.2	1.6 ± 0.2	1.8 ± 0.4
**Lnkb2**	1.0 ± 0.0	1.4 ± 0.2	5.2 ± 0.6	6.8 ± 1.2	17 ± 3
**TF3B5**	1.6 ± 0.4	1.1 ± 0.1	1.4 ± 0.2	1.8 ± 0.4	1.0 ± 0.0
**C3**	7.4 ± 1.8	9.5 ± 1.4	9.6 ± 1.3	2.2 ± 0.8	3.4 ± 0.6
**9E10.3**	42 ± 3	79 ± 4	13 ± 2	24 ± 3	33 ± 3

[1]The one million random sequences in each case are all the same size as the antigen sequence used.

antigen. In each case the full model including weighting, shifting and removal of compositional binding was used. Table S4 shows the sequences used and Table S5 shows the resulting ranks out of 1 million random sequences of that length. While there is some variation in rank with sequence length, it is modest, with some of the predictions being slightly worse with sequences longer than 7 and some slightly better.

Mutation Substitution Matrices. Fig. S6 is similar to Fig. 2 in the main text, showing the predicted binding values of every single amino acid substitution of the cognate sequence of each

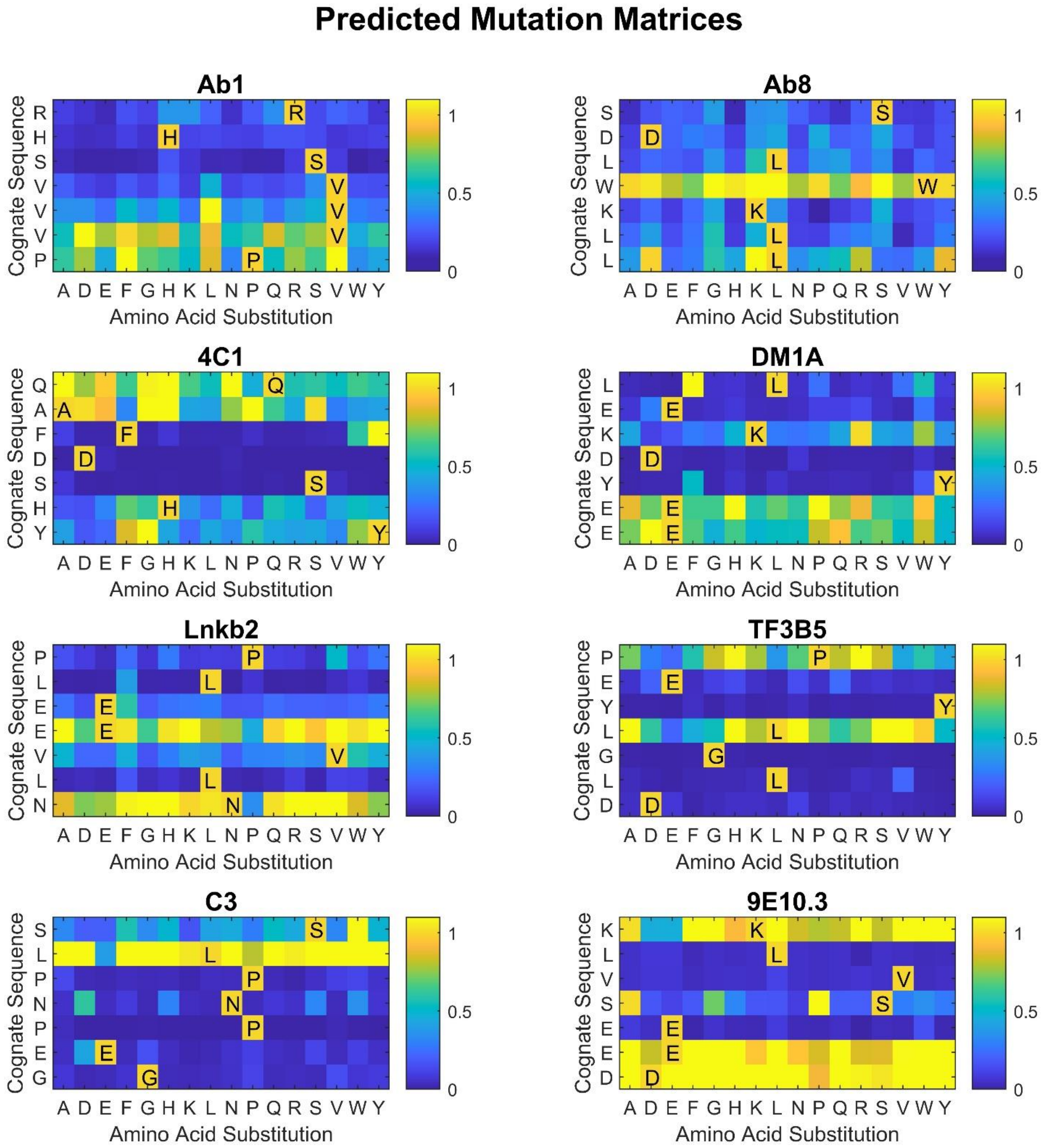

Figure S6. Predicted mutation matrix for each of the mAb cognate sequences. The y-axis is the cognate sequence and the x-axis is the amino acid used for substitution. The color is proportional to the relative binding predicted by the neural network (see color bar). In each case, the values were normalized to the predicted binding of the cognate sequence. Note that any sequence with a value of 1.1 times the binding value of the cognate sequence is yellow. This allowed better resolution of values less than 1. See Fig. 2 in the main text for details.

of the mAbs. Fig. 2 showed only four of these and compared the predicted and measured results. Here all eight mAb predictions are shown (no measured values). The cognate reference sequences used here are the same as those in Table 2 of the main text, but not always identical to Fig. 2 of the main text. For details, see the main text.

Reducing the Total Number of Sequences in the Training Set Randomly. Fig. S7 shows the change in rank of the 7mer cognate sequence in a million random 7mer sequences as the number of sequences in the training set is reduced. In each case a random subset of sequences was chosen and the full model (weighting, shifting and compositional binding removal) was used (see the final column of Table 2 in the main text for comparison). The results are described in the main text.

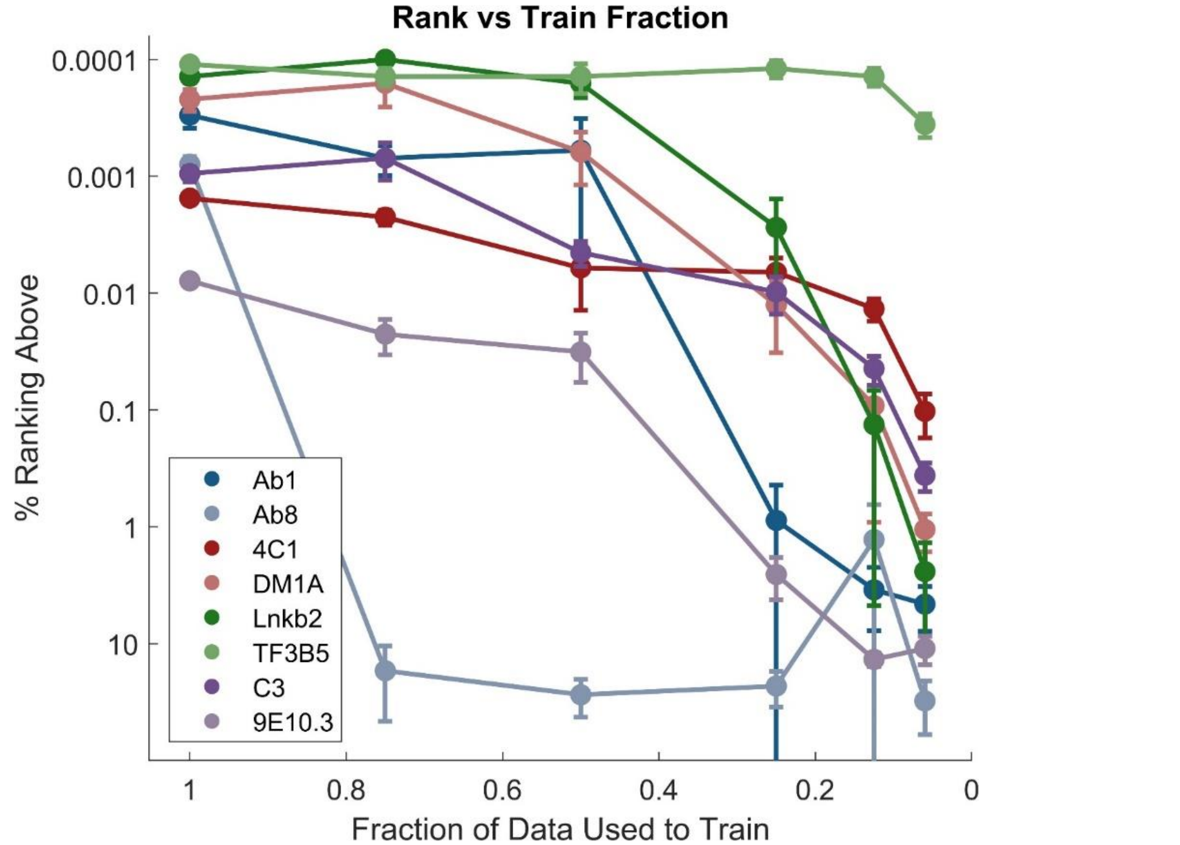

Figure S7. Ranks of the 7mer cognate epitope (Table 1 in main text) in 1 million random sequences using training sets of array peptides in which a random subset of peptides was used. The Y axis is the percent rank, with 0.0001% signifying rank 1 in a million. The X axis is the fraction of the ~122,000 peptide sequences on the array used, randomly chosen. The points and error bars represent the average and standard error of 5 randomly chosen training sets each evaluated by 12 randomly initiated models.

Removing Similar Sequences from the Array Peptide Library Before Training

An important question is how strongly the models created depend on the similarity of a few sequences to the mAb cognate sequence. To test this, starting with the cognate 7mer sequence (Table 1 in the main text), first only peptides with no more than 6 amino acids in common were used in training (note there were none with all 7 so <=6 is the full set), then only those with no more than 5 in common, etc. down to only those with no more than 2 in common. The definition of "in common" used was having the right sequence and the right relative position, but occurring in any register within the 7mer (including registers where the cognate sequence and the comparator sequences only partially overlap). Thus, for SDLWKLL, the sequence RGRWADLGK would be considered to have 3 in common with the cognate (XXXXXDLXK) because the sequence order and spacing of three amino acids match, even though the register is shifted. Table S6 shows how many sequences were removed to achieve each reduced training set. Table S7 shows the ranks that resulted using the full model (weighting, shifting and compositional binding) trained on each reduced peptide set. Fig. 4 in the main text presents this graphically.

Table S6: Number of Peptides Removed to Reduce Similarity

	Number Sequences Removed to Achieve:				
**mAb**	**≤6 matches**	**≤5 matches**	**≤4 matches**	**≤3 matches**	**≤2 matches**
**Ab1**	0	0	3	150	3533
**Ab8**	0	0	7	257	4386
**4C1**	0	0	3	156	4005
**DM1A**	0	1	4	240	4688
**Lnkb2**	0	0	8	222	4381
**TF3B5**	0	1	10	334	6134
**C3**	0	1	26	543	7395
**9E10.3**	0	0	33	701	9075

Table S7: Ranks After Training on Peptide Sequences with Reduced Similarity to the Cognate

		Ranks for the best 7mer Tile with less than:				
**mAb**	**Epitope Sequence**	**≤6 matches**	**≤5 matches**	**≤4 matches**	**≤3 matches**	**≤2 matches**
**Ab1**	RHSVVVP	3.0 ± 0.7	2.2 ± 0.4	4 ± 0.4	30000 ± 4000	22,000 ± 1,300
**Ab8**	SDLWKLL	7.9 ± 1.2	10 ± 3	320,000 ± 20,000	290,000 ± 8,000	380,000 ± 30,000
**4C1**	QAFDSHY	15 ± 1.5	12 ± 1	13 ± 3	14 ± 1	210 ± 30
**DM1A**	LEKDYEE	2.2 ± 0.5	4.2 ± 0.9	82 ± 5	1000 ± 110	160,000 10,000
**Lnkb2**	PLEEVLN	1.4 ± 0.2	1.8 ± 0.4	3.6 ± 0.7	1.2 ± 0.2	21,000 ±4,000
**TF3B5**	PEYLGLD	1.1 ± 0.1	1.0 ± 0.0	1.0 ± 0.0	6.0 ± 1.1	60,000 ± 6,000
**C3**	SLPNPEG	9.5 ± 1.4	8.4 ± 1.0	14 ± 1	57 ± 7	4500 ± 300
**9E10.3**	KLVSEED	79 ± 4	63 ± 4	160 ± 20	860 ± 170	200,000 ± 5,000

Data and Algorithm Availability
Dataset. The averaged data for all 8 mAbs is available on Zenodo https://zenodo.org/records/12510566. The data is in an excel spreadsheet, 'Averaged_Array_Data.xlsx'. Column 1 contains the sequence corresponding to that row of data. Column 2 is either 0 or 1, with 1 meaning that the sequence and data in that row is for a mAb cognate sequence purposely included on the array. These sequences are removed by the algorithm prior to neural network training. The remaining 32 columns are the data for each mAb at each concentration (each value is the average of 4 measurements). The top row contains the name of the mAb and the second row contains the concentration. The subsequent values are the average measured binding values. Note that there are more values here than were used in the analysis because all sequences over 11 residues in length were excluded in the analysis. The values given are binding values in counts. The detector saturated at 65536 counts. The data for each of the four replicates averaged is also included as "Dataset_repx.xlsx" where "repx" is rep1 through rep4. These files have the same format as the averaged file.

Algorithm. The Matlab script is available on Zenodo as well https://zenodo.org/records/12510566 ('mAb_sequence_binding_relationship_updated.m') containing all the functions at the end. At the top of the script there are a series of values that can be varied:

Table S8: User defined variables in the Matlab Script

Variable	Options (Default)	Description
current_mAb	Ab1, Ab8, 4C1, DM1A, Lnkb2, TF3B5, C3, 9E10.3	The name of the mAb to analyze
Data_file	(Averaged_Array_Data.xlsx) or Dataset_repN.xlsx	The name of the data file
num_rep	User defined (12)	The number of neural network models averaged
numrand	User defined (1000000)	The number of random sequences predicted
num_layers	User defined (2)	The number of hidden layers
num_nodes	User defined (250)	The number of nodes/layer
miniBatchSize	User defined (1000)	The number of sequences analyzed per mini batch
MaxEpochs	User defined (20)	The number of training Epochs
InitialLearnRate	User defined (0.002)	Learning rate during training

LearnRateDropFactor	User defined (0.9)	Rate at which the learning rate drops during training
shift	User defined (5)	The number of different registers of each sequence in the input vector used during training. 0 means no register shifting
weight_high_binders	true/false (true)	Determines if weighting of high binding values is used
maxdensity	User defined (300)	If weighting used, this is the number of sequences per bin after copying
maxcopy	User defined (100)	If weighting used, this is the maximum number of any one sequence
conc2compare	0.125, 0.5, 2, 8 (8)	If weighting used, this is the concentration used to set the number of copies of high biding sequences
EndMarkers	true/false (true)	Determines if end markers are used
adjust_saturated_points	true/false (true)	Determines if saturated points are extrapolated based on concentration
Composition_fit	true/false (true)	Determines if compositional binding is subtracted from the data before training the model
ExcludeLong	true/false (true)	Determines if the longest sequences are excluded from the training
maxlen	8-13 (11) shorter than 8 would remove too much data	If longest sequences are excluded, this is the maximum length used

The script is set up to run as long as the data file is in the same directory as the script. Note that this script was developed under Matlab release 2022a, though it should work on a range of release versions.

Literature Cited

1 Moore, C. *et al.* A unified peptide array platform for antibody epitope binning, mapping, specificity and predictive off-target binding. *bioRxiv*, 2022.2006.2022.497251, doi:10.1101/2022.06.22.497251 (2022).

2 Eilers, P. H. C. & Goeman, J. J. Enhancing scatterplots with smoothed densities. *Bioinformatics* **20**, 623-628, doi:10.1093/bioinformatics/btg454 (2004).
